# National health governance, science and the media: drivers of COVID-19 responses in Germany, Sweden and the UK in 2020

**DOI:** 10.1136/bmjgh-2021-006691

**Published:** 2021-11-17

**Authors:** Claudia Hanson, Susanne Luedtke, Neil Spicer, Jens Stilhoff Sörensen, Susannah Mayhew, Sandra Mounier-Jack

**Affiliations:** 1 Global Public Health, Karolinska Institutet, Stockholm, Sweden; 2 Disease Control, London School of Hygiene & Tropical Medicine, London, UK; 3 Institute for risk and disaster reduction, University College London, London, UK; 4 Gesundheitsamt Nuremberg, Nuremberg, Germany; 5 Faculty of Public Health and Policy, London School of Hygiene and Tropical Medicine Faculty of Public Health and Policy, London, UK; 6 School of Global Studies, University of Gothenburg, Goteborg, Västra Götaland, Sweden; 7 London School of Hygiene and Tropical Medicine Faculty of Public Health and Policy, London, UK

**Keywords:** COVID-19, health policy, public health

## Abstract

The COVID-19 pandemic is an unprecedented global crisis in which governments had to act in a situation of rapid change and substantial uncertainty. The governments of Germany, Sweden and the UK have taken different paths allowing learning for future pandemic preparedness. To help inform discussions on preparedness, inspired by resilience frameworks, this paper reviews governance structures, and the role of science and the media in the COVID-19 response of Germany, Sweden and the UK in 2020. We mapped legitimacy, interdependence, knowledge generation and the capacity to deal with uncertainty.

Our analysis revealed stark differences which were linked to pre-existing governing structures, the traditional role of academia, experience of crisis management and the communication of uncertainty—all of which impacted on how much people trusted their government. Germany leveraged diversity and inclusiveness, a ‘patchwork quilt’, for which it was heavily criticised during the second wave. The Swedish approach avoided plurality and largely excluded academia, while in the UK’s academia played an important role in knowledge generation and in forcing the government to review its strategies. However, the vivant debate left the public with confusing and rapidly changing public health messages. Uncertainty and the lack of evidence on how best to manage the COVID-19 pandemic—the main feature during the first wave—was only communicated explicitly in Germany. All country governments lost trust of their populations during the epidemic due to a mix of communication and transparency failures, and increased questioning of government legitimacy and technical capacity by the public.

**Key questions**

**What is already known?**

- Governments in Europe reacted in different ways to the COVID-19 crisis; the Swedish exception has raised debate.
- Little is known, however, how differences in the pandemic handling related to government structures, the role of academia and the communication with the public.

**What are the new findings?**

- Germany, Sweden and the UK responded in a very different way in line with the (i) pre-existing societal and academic culture, (ii) the existence of trusted academic advisory boards and (iii) the ability to manage and leverage diversity allowing broad academic involvement and societal debate.
- Germany leveraged diversity and inclusiveness and allowed a broad societal debate, but this overwhelmed the population in the second wave.
- Sweden feared different views: the government instead delegated the handling of the pandemic to the Public Health Agency.
- The UK leveraged its strong academic structures, but the public was left with confusing and rapidly changing public health messages.

**What do the new findings imply?**

- Pandemic preparedness will need to go beyond traditional approaches to preparedness within the health sector and state emergency function.
- Strong pre-existing, trusted and functional academic and public advisory bodies that can support decision-making, evidence creation as well as communication with and engagement of the public may increase resilience—but these structures can only be fully leveraged if politicians and the media are able to provide them space.

## Introduction

The COVID-19 pandemic is an unprecedented global crisis. Reported cumulative global cases and deaths were 83 and 1.8 million, respectively, at the end of 2020, and Europe accounts for around one-third of global cases and deaths.^1^ European countries were insufficiently prepared when a large COVID-19 outbreak in northern Italy first became evident.^2^

Governments were forced to respond to a crisis characterised by many uncertainties, especially relating to levels of presymptomatic transmission^3 4^ and infection-fatality rates.^5^ Interventions to address the epidemic were first based on practices used to curb common influenza epidemics including handwashing. The use of masks was adopted, but only hesitantly at first. When the second wave hit Europe in autumn 2020, uncertainty was substantially reduced. In early November, consensus emerged on infection-fatality rates, with estimates ranging from 0.4%^6^ to 0.8% across European countries,^7 8^ confirming higher levels than in typical influenza epidemics.^9^ There was broad consensus at that time on the importance of transmission through smaller airborne droplets (aerosols), the importance of presymptomatic and asymptomatic infection transmission^10^ and more clarity about the role of infection and transmission in children.^11 12^ In the midst of the second wave, news about the imminent availability of effective vaccines was released.^13^ Soon after, new mutations discovered in the UK and South Africa curtailed hopes that the pandemic could be tackled within a few months; again this raised uncertainty.

Government decision-making in such a crisis, particularly when scientific uncertainty is high, demands capacity and legitimacy to protect citizens as well as health systems.^14^ Concepts of health systems resilience—the capacity to adapt and respond to shocks—have been discussed and framed in recent years. This includes critical consideration in reducing the negative and often unequal effects on health that can result from crises.^15^ Discussions about system resilience emerged during and after the 2013–2016 West Africa Ebola outbreak. That time evidence was created—although often ignored—on the importance of health systems being adaptable to sudden crises,^16 17^ contributing to thinking on how systems could also adapt to longer-term challenges such as climate change.^8 18^ Subsequently, a larger body of evidence has been gathered to better conceptualise and refine the concept of health system resilience, and studies are beginning to use this concept to analyse systems responses to outbreaks.^19 20^ Few have applied resilience as a lens for the analysis of governance and government decision-making in crises to review the relevance of the domains.^21^ Yet, the COVID-19 crisis highlights the importance of governance of the health systems *and* health.^22^ The importance of assessing processes, including communication, building legitimacy and creating knowledge in the population, in addition to focussing on outcomes such as morbidity and mortality, cannot be overemphasised.^23^

After over 1 year into the COVID-19 pandemic, several scholars have started to rank country performance. Within Europe large differences in excess mortality has been described for 2020.^24^ The Bloomberg Resilience Score takes a more holistic approach and includes in addition to mortality also social freedom, vaccination and other indicators describing the ability to go back to noramality.^25^ The ranking of better and worse performers raises the question of factors and processes which made countries to be more or less successful. Those countries, including Sweden and the UK, which scored highest on Global Health Security Index—a six-category score encompassing aspects of detection and reporting, rapid response and health systems readiness—did not demonstrate an effective response, raising questions about aspects of crisis readiness that were missed in the score.^26 27^

To support the further conceptualisation of potential factors which increase epidemic preparedness and increase societal and health systems resilience we use the Blanchet *et al* resilience framework to better understand the COVID-19 response and particularly governance and leadership, the link to science, and how this shaped communication with populations leading to enhanced or damaged inclusion and trust. Further, we aimed to contribute to theoretical reflections on governance and resilience and how to better frame necessary processes and decision-making to strengthen future crisis management and pandemic preparedness.^28^

We selected the three countries on the European continent of Germany, Sweden and the UK because the (i) were being hit at virtually the same time in 2020 but (ii) adopted very distinct approaches to the first and second wave. Germany was early characterised as well-performing.^29^ The Swedish exceptionalism was unique in Europe and has raised much international debate.^30^ The UK has a strong academic public health tradition and UK based researchers were publishing important background papers,^31 32^ yet COVID-19 mortality rates were high and its response to the first wave was heavily criticised. COVID-19 mortality rates differ strongly between the three countries in the first and second wave and differences are still seen.^33^

## Methods and conceptual framing

**Figure 1:**
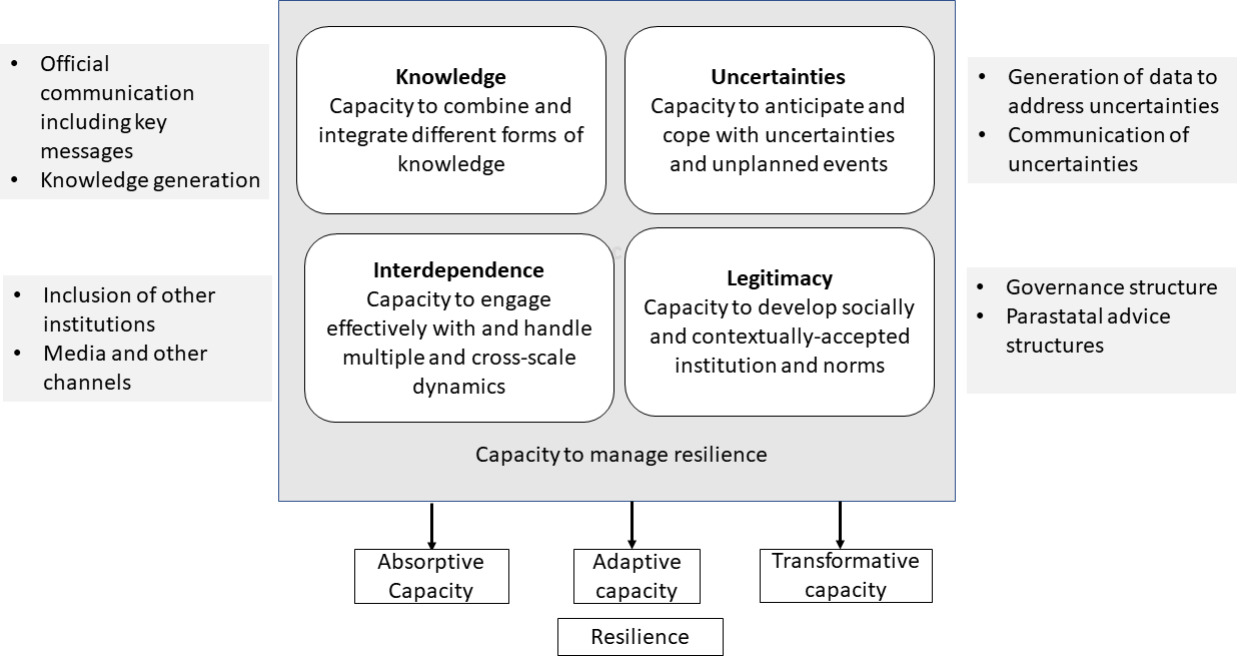
Resilience framework to assessing government responses to COVID-19 (adapted from Blanchet *et al*^34^).

We analysed governance, policies and communication of the governments of Germany, Sweden and the UK, informed by the resilience framework of Blanchet *et al*^34^ with additional adjustments relating to cross-cutting dimensions as hypothesised by Hanefeld *et al*.^15^ The domains within our framework (figure 1) are supported by governance scholarship^14 35^ and are particularly relevant for thinking about health crisis management, and hence for conceptualising key issues that have arisen during the COVID-19 pandemic. In particular the framework reflects the importance of (i) legitimacy of governance and decision-making,^13^ (ii) knowledge creation and communication, particularly when there is scientific uncertainty and (iii) collaboration with as well as interdependence between the community and other actors including scientists and the media.^36^

We populated the original elements with information from the three countries. Specifically, we mapped legitimacy, interdependence, knowledge generation and the capacity to deal with uncertainty. Our analysis describes the capacity of these countries to manage the crisis in 2020.

The paper is based on a systematic document and policy analysis.^37^ However, while we sought to directly compare the three countries, in practice it was necessary and appropriate to adopt a flexible approach when identifying the documents that we drew on. This was because the sources of information on COVID-19 varied substantially between the countries. Also, the nature of the pandemic, the policy resources and sources of information changed very rapidly throughout 2020.

Much of the information we used was drawn from government and public health Agency websites of countries’ public health agencies and governments: the Robert Koch Institute, Germany,^38^ the Public Health Agency (PHA), Sweden,^39^ and the Coronavirus (COVID-19) page of the UK government (Gov.UK).^40^ We also included mass media websites. A list of websites searched and keywords used is provided in online supplemental web annex 1. We analysed the policies of the countries in relation to each domain of our framework. Information was cross-checked with already published work and carefully referenced to ensure transparency.

10.1136/bmjgh-2021-006691.supp1 Supplementary data

We reconstructed the timeline of events and interventions from government websites and mass media and extracted data on the 14-day rolling average of cases, deaths and SARS-CoV-2 testing from the European Centre for Disease Prevention and Control (ECDC)^33^ except for the UK testing data for which we extracted data from the government homepage of England, Wales, Scotland and Northern Ireland and calculated weekly rates per population.^40^ We used the COVID-19 Government Response Stringency Index,^41^ to assess the strength of the government intervention in each country. We used the YouGov COVID-19 tracker to summarise levels of trust by plotting the answers to the question of whether respondents agree that the government handled the coronavirus ‘very’ and ‘somewhat well’.^42^ We imputed missing data points using the *impute* command in Stata V. 16.

## Results

### Timeline of the pandemic and the response

**Figure 2:**
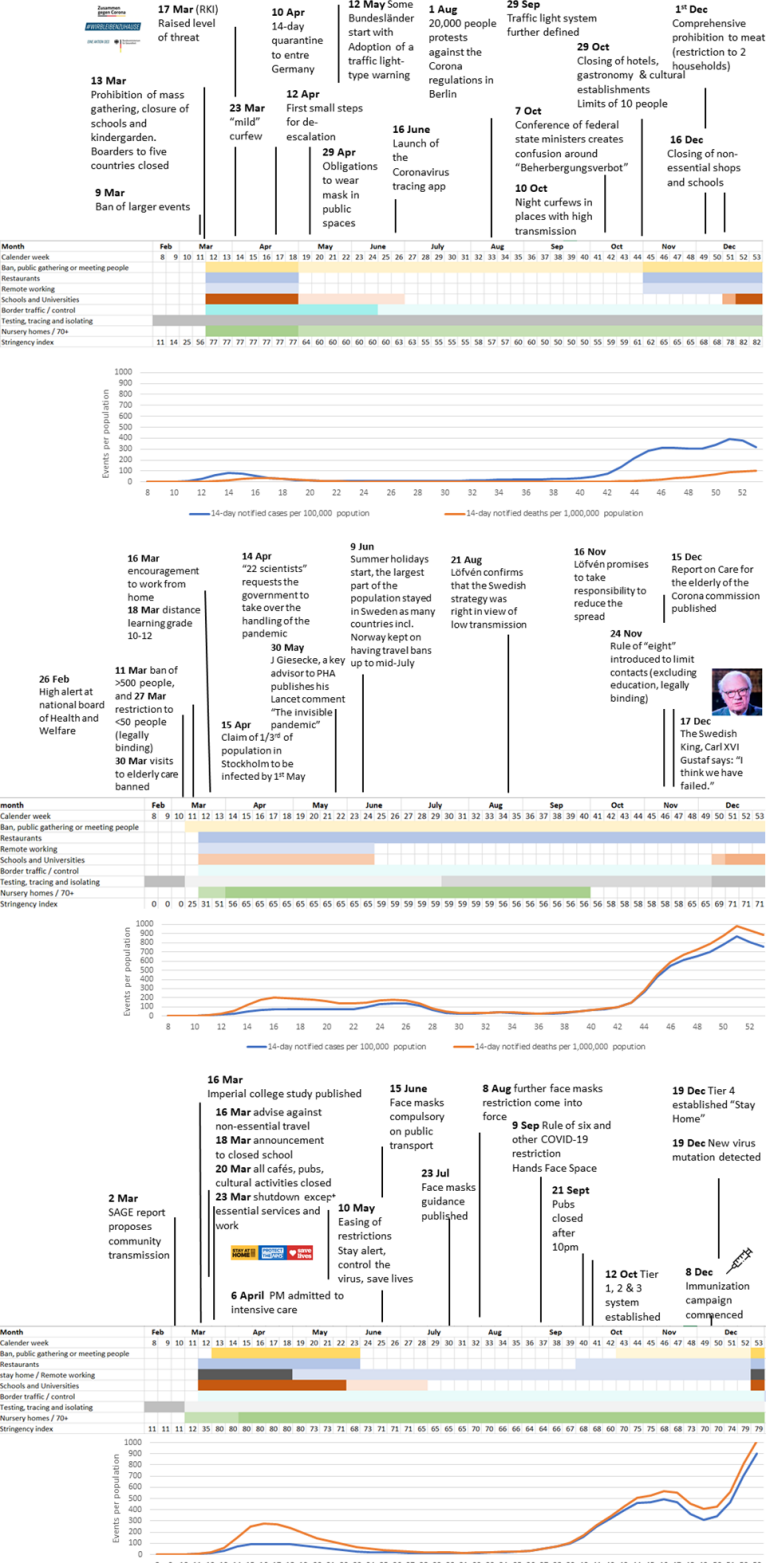
(A–C) Timeline of responses in Germany, Sweden and the UK (February–December 2020) (online supplemental web annex 2 references to timeline).

All three countries detected their first cases of SARS-CoV-2 in January 2020 which triggered their first responses, but they only started to act more decisively when community transmission became apparent in the three countries in early March (figure 2A–C, online supplemental file 4).

10.1136/bmjgh-2021-006691.supp4 Supplementary data

10.1136/bmjgh-2021-006691.supp2 Supplementary data

Both Germany and the UK responded on 23 March with national restrictions, although of different intensity. Educational, leisure and cultural facilities were closed in both countries. The UK instituted strict ‘stay-at-home’ orders, while Germany allowed people to meet outside. In contrast, Sweden’s public life remained less interrupted. Schools remained open, although the last three grades moved to distance learning. Also, large events were banned. Given the fast and more restrictive reaction of other Nordic countries, the PHA’s strategy came in for early criticism by some.^43^

Cases started to decline in mid-April in Germany and the UK at which point measures were relaxed and public life partly restored, and schools were reopened. In contrast, there were only minimal changes in Sweden where less stringent measures remained including a ban of larger events. Germany and the UK had very low numbers of cases of just 6 (Germany) and 12 (UK) over a 14-day rolling average per 100 000 population in June and July 2020, while in Sweden cases were never reduced below 25 infections in 14-days per 100 000 population. Schools in all three countries reopened in autumn 2020 for the new school year.

In the UK, the second wave started to become apparent in late September, which was about 10 days earlier than in Germany and Sweden. In Germany, a *circuit breaker*, including closing cafes, restaurants and cultural activities was implemented. Cases stabilised but did not decline, which lead the government to close non-essential shops and institute home-schooling by mid-December. In Sweden, the increase in cases in October took the population and government by surprise, given that the PHA had repeatedly stated Sweden would be less severely hit because of the larger spread in the spring of 2020.

In the UK, and similarly in Germany, softer control measures were initially introduced during the second wave. At the start of December, relaxation measures were implemented until a new lockdown was enacted later that month when new variant strains started to emerge and spread in the build up to Christmas.

## Interdependency and legitimacy

### Governance set-up

Throughout the first and second wave, in both Germany and the UK, the elected federal and national governments led the response. In contrast, the Swedish national government, although formally responsible, were little visible in the public and rather entrusted leadership to the PHA. These institutions had different levels of public and legal legitimacy and different sets of interdependencies (table 1). In Germany and the UK, emergency powers were enacted in March 2020 through Infection Protection Acts. In Sweden, a COVID-19 emergency law was passed only in January 2021.

**Table 1:** Legitimacy and interdependence: governance set-up, and inclusion of other bodies

	Germany	Sweden	UK
Health governance structure	The federal structure with democratically legitimate ‘Bunderländer’ take key responsibility in line with constitution.^90^ A crisis task force, the ‘Corona Kabinett’ jointly led by the German Federal Ministry of the Interior (BMI) and German Federal Ministry of Health (BMG) gathers all ministry-specific competences.^91^ Provision of healthcare is under the auspices of the ‘Bundesländer’ and governed by the devolved healthcare system.	Two constitutionally legitimate crisis management structures—one under the Ministry of Interior and a group for strategic coordination (strategisk samordning) are foreseen but were not activated.^92^ Government delegated the handling of the pandemic largely to its state Public Health Agency (PHA). It remains unclear if constitutional restriction, such as the *ministerial rule* or other bills restricted government involvement.^93 94^ The provision of healthcare and the operationalisation of the response is devolved to 21 county councils and regions.^83^	The government is ultimately responsible together with the 4 UK governments’ chief medical officers (CMOs). Implementation and the institution of public health policies was devolved to Public Health England (PHE) as well as the Public Health bodies in Scotland and Wales and the Public Health Agency of Northern Ireland. Cabinet Office Briefing Room A (COBRA), the crisis cabinet met unfrequently during the crisis, thus leaving weak coordination between nations. The provision of healthcare is organised by the National Health Services (NHS) with their public health departments.
Laws passed	In Germany, a revision of the 2001 Infection Protection Act (*Infektionsschutzgesetz*, IfSG) was passed by the parliament on 27 March and again on 18 November.^95^	In Sweden, the Infectious Diseases Act 2004: 168 gives key responsibility to the individual and may limit government response.^96^ A COVID-19 emergency law passed in January 2021 provides the government with stronger emergency power^93^ contradicting earlier explanations that the government was not able to pass a law providing emergency power.	In the UK, the Coronavirus Act 2020 gave the government emergency power over parts of the NHS, social care, schools, police, the border force, local councils, funerals and courts.^97^ The act received royal assent on 25 March 2020.
Statal and parastatal advice structures	Robert Koch Institute (RKI), a federal institution, is charged with the nationwide health monitoring. It collects and interpret epidemiological data laid down in the Infektionsschutzgesetz (IfSG). The RKI provides advise to the government. The Robert Koch Institute has no mandate to decide on strategies.^38^	The PHA advised and released recommendation in relation to direct health matters. The legitimacy of its recommendations, for example, against the closing of public life and against closing schools is unclear.^98^	Scientific advisory Group for Emergencies (SAGE) committees provide independent scientific advice, but this is non-binding. Membership of SAGE and meeting minutes was made public since June 2020.^99^ PHE was established in 2013 and is an executive agency of the Department of Health and Social Care, and a distinct organisation with operational autonomy. Its role is to protect and improve the nation’s health and well-being and reduce health inequalities.
Inclusion of academia	A multitude of universities and research institutions involved throughout to inform the government and the public. Research institutions conceived advisory boards to inform the government, among them the Helmholz Institute.^100^ Deutsche Akademie der Naturforscher Leopoldina - Nationale Akademie der Wissenschaften.^101^ Ethics advisory board.^102^	The PHA agreed on an advisory board, but no minutes or meeting schedules are published.^46 91^ A multi-professional group formed in April 2020 has been active in publishing in newspapers, in preparing educational talks and summarising evidence.^103^ The group has no official mandate.	SAGE is a standing committee. It held twice-weekly meetings since January 2020. SAGE relies on external science advice and on advice from expert groups. During the COVID-19 pandemic this included the New and Emerging Respiratory Virus Threats Advisory Group (NERVTAG), the Scientific Pandemic Influenza Group on Modelling (SPI-M) (Department for Health and Social Care), Independent Scientific Pandemic insights Group on Behaviours (SPI-B) and others. The so-called alternative SAGE includes 18 academics which post live broadcasting and alternative reports and guidance.^104^ They receive a good amount of media coverage but have no official mandate.
Inclusion of other institutions	The German Ethics committee was consulted in regular intervals to provided ad-hoc advice, such as on restrictive measures as well as also vaccination, etc.^105^	End of June 2020 a Corona commission was launched, an independent expert committee to assess the national strategy.^106^ The first report on care for the elderly was release mid-December 2020.^107^	No other institution or link to lay persons.
Media	Corona becomes part of the daily key public news. The public television channels have been including debate sessions of each about 5 hours per week. These debate sessions allowed a broad discussion between scientists, politicians, philosophers etc. Different views were included and a high-level understanding of the underlying principles as well as the challenges were thus made open to the public.	Daily, later bi-weekly television broadcasting with the state epidemiologist presenting on most occasions. A total of 200 press conferences have been held.^108^ Media largely supported the national strategy, with limited critical questions to the PHA.^73^ Media have been overwhelmingly providing the PHA with space to outline their views.	Daily government briefings, which include a range of ministers, the CMO, and the prime minister. These are broadcast live. Complemented by pre-recorded statements from the prime minister.
Other media channels	Since the 26 February a podcast where one of the leading virologists Christian Drosten, explains each weekday for about 40 min what is known in plain language and gives thus a large number of people a good understanding of what is known and what is not known.	no any	There is a wide engagement of universities. Alternative YouTube broadcastings by scientists are available—although they have not been formalised. LSHTM has been notable in hosting open talks which the public can access.

As a federal state, Germany installed a coordinating and decision-making body headed by Chancellor Merkel which included all heads of its regional states. This allowed strong locally adapted action. In Sweden, no national crisis management programme was activated. The UK, as a union of four nations, initially pursued aligned unified approach through ministerial implementation. This gradually diverged in the second wave, with responses more strongly led by the devolved administrations of England, Scotland, Wales and Northern Ireland. Hence, the UK nations increasingly implemented their own specific measures and timetables based on the local epidemiological situation and political context.^44^ For example, Wales instituted a *circuit breaker* during the school holidays, Scotland closed the borders before Christmas and England introduced a three-tiered system of restrictions.

#### Interdependency: inclusion of scientific bodies and seeking advice

Interdependence between the policymaking structures and academia was established in different ways. Germany relied on existing legal structures such as its parastatal Robert Koch Institute, the Ethics Advisory Board and its wide network of independent research institutions. Consultations with other scientists were demand-based and not part of a predefined scheme.

The UK used and expanded on its pre-existing national scientific committee, the Scientific Advisory Board for Emergencies (SAGE). SAGE became very influential: the lockdown implemented on March 2020 was said to have been a direct response to a modelling paper provided by a member of SAGE.^31^ SAGE was initially contested because of a lack of transparency of its membership, but remained influential because of its wide academic membership and remit and the role of its members in directly communicating evidence to the media and the wider public.

While the UK had traditionally strong independent public health agencies, Public Health England (PHE) had already lost much of its autonomy and independence with the demise of its predecessor, the Health Protection Agency. The present government opted for a stronger inclusion of the private sector and shifted the testing and tracing strategy and delivery away from PHE.^45^ The prime ministers’ office was also preoccupied with Brexit and other priorities and appeared unprepared to listen to academia.^32^

In Sweden, the PHA, in line with their mandate, led on health protection-related policies on behalf of the government. A formal advisory board was instituted in April 2020 after criticism of the dominance of PHA emerged, but no minutes or meeting agendas were made publicly available.^46^

#### Interdependency: media

In Germany, print media, public television and radio played a large role in communication and information sharing. Both public TV channels ARD and ZDF, provided ample space for intensive information and debate of several hours per week (online supplemental file 3). Mainstream as well as critical voices opposing the main government strategies were invited. The inclusion of multiple voices, including virologists and epidemiologists, together with politicians and other members of civil society, economists, political scientists, philosophers and ethicists enabled the public to follow the complexity of decision-making. The innovative format of a daily podcast with Christian Drosten from Charité was launched on 26 February 2020 and is still running with over 50 million listeners^47^ and has received prizes.^48^

10.1136/bmjgh-2021-006691.supp3 Supplementary data

In Sweden, daily press conferences gave the PHA ample space to report. Public television provided very few opportunities for scientists to contribute to the national conversation. Only seven debates were included in the public television. However, there was debate in the daily newspapers. The UK, however, had extensive COVID-19 media coverage, across all types of media and surveys showed that the majority of the public relied on the mass media for information about COVID-19.^49^

### Knowledge generation and dealing with uncertainty

#### Knowledge: generation of data

Data needed for monitoring the pandemic were provided by the public health agencies in Germany and Sweden. Daily or weekly reports were made public. Germany scaled-up testing rapidly during the first wave, although hit capacity limitations similar to those of Sweden and the UK in the second wave (table 2, online supplemental file 4). In the UK, the Test, Track and Trace system was outsourced to the private sector from April 2020. This led initially to there being insufficient sites and poor geographical distribution with insufficient data to ensure locally targeted approaches with local involvement.^50^ However, the UK’s academic public health structures with government research funding made it possible within a short time to set up national population-based surveys such as the SARS-CoV-2 Infection and Immunity Survey and the Covid Symptom Study. Neither Germany nor Sweden had such established surveillance systems although there were smaller, local surveys.

**Table 2:** Knowledge generation and dealing with uncertainties

	Germany	Sweden	UK
Key structures	The government very quickly scaled-up testing for COVID-19 within the public health system. Early studies alluded to the presymptomatic and asymptomatic spread.^109 110^ The test, trace and isolate system managed by the public health system which received major financial and personal support for its activities. Federal governments supported generation of evidence through funding, such as the assessment of infection-fatality rate after a superspreading event.^105^	Testing for COVID-19 reduced to those ill in response to severe testing shortages in March. Community testing was scaled-up mid-May. Testing is provided within the public healthcare systems as well as by private companies. Hospitals employed teams for tracing contacts. At primary care levels the responsibility lies also within the primary care structures. However, the largest part of the testing is done as ‘home-testing’ and the patient is to a high degree responsible for tracing.	Testing, originally planned to be managed by the public system, was suspended in March 2020, and only provided to those being admitted to hospitals due to capacity problems. Since April 2020, tracing and testing is done primarily by private firms. Some local authorities developed their own test and trace system in response to this.^50^ During the first wave local authorities had no access to data.^111^ Directors of Public Health started to receive postcode-level data on infections in their area only from 24 June 2020.
Laboratory surveillance of identified infections	Daily reports in German and English since 4 March 2020.	Daily numbers and weekly reports in Swedish.	Daily reports.
Deaths	Daily reports as above.	Daily numbers, weekly reports in Swedish.	Daily reports of death within 28 days of positive tests.
Surveys of infection in the community	RKI-SOEP study a longitudinal design including 30 000 people/15 000 households. Several smaller and larger studies, for example, the Kupferzell study is investigating one of the early hotspots. In addition, there are several studies independently from RKI on schools and preschools, etc.	Four population-based surveys, August and September with each 2500 part.	COVID-19 Infection Survey (ONS), established in April 2020, repeated cross-sectional (once-a-month) population-based survey intervals.^112^ REACT, repeated cross-sectional survey (5+ years) in samples of 100 000 participants.^113 114^
Messaging	The key messages in the pandemic were not changed but complemented throughout the pandemic. To support the national curfew in March and April, the slogan ‘We stay home’ was circulated since 18 March.^115^ First messages included distance and hygiene. Wearing a non-medical mask was added in May. With the launch of the CoronaApp 16 June and the evolving evidence that the disease is transmitted by small droplets (aerosols) the logan was further expanded to encompass the opening of windows for air circulation. 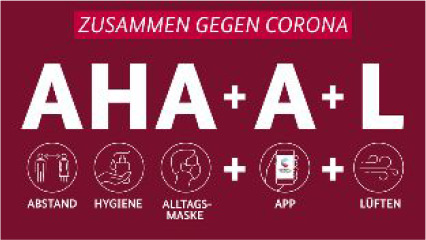 **Translation:** A Abstand (distance), H Hygiene, A Alltagsmaske (non-medical masks)+A App (Corona-App)+L Lueften (air circulation).	The main messages remained unchanged throughout 2020. 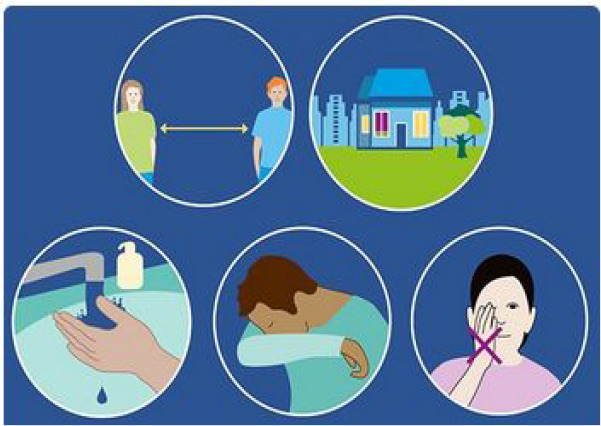 The key message of ‘stay home when you are sick’^116^ was complemented by ‘wash your hands and the rule of 2-metre distance’. Asymptomatic/presymptomatic transmission was little recognised.^117^ Masks were recommended only in 2021 and only in limited settings such as health facilities.	Messages and slogans changed throughout 2020, which was perceived as confusing by the population.^71^ Stay at home, protect the NHS, save lives (March 2020). 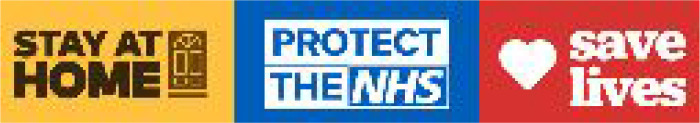 Stay alert, control the virus, save lives (May 2020). Hands, face, space—wash hands, cover face, make space (July 2020). 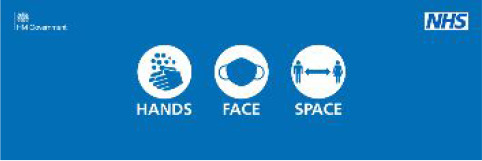 Rule of six (September 2020). ‘V-Day’ (December 2020).
Communicating goals	(i) Reducing morbidity and mortality in the population, (ii) caring for sick people, (iii) maintaining essential public services and (iv) providing reliable and timely information for political decision-makers, specialists, public and the media.^118^	(i) Protect senior and/or vulnerable citizens and (ii) slow down the spread of the virus so. The strategy was communicated as lowering the epidemic curve.^53 119^ It remains unclear (and debated) if reaching herd immunity was an underlying goal.^61^ Emails released under the public transparency law and public talks and written comments by Giesecke, a key advisor to the PHA, suggested that allowing the infection to spread slowly to establish immunity may have been an underlying philosophy.^120 121^	Pandemic action plan listed 3 phases—contain phase; the delay phase; the research phase and the mitigate phase—phased response.^122^ This plan has not been updated. Specific plans have complemented this initial plan including, for example, the UK COVID-19 vaccines delivery plan and the Test and Trace Business Plan.
Communicating goals and data	R0 below 1 as well as a cut-off value of 50 infections in a week per 100 000 population. The RKI informed the public throughout the epidemic on the homepage (German and English), regular (typically biweekly) press-conferences on figures of infection, transmission and deaths. Sex and age distribution. Regular reporting on infections in home for elderly and in educational institutions.	No clear goal or target formulated. Daily press conferences, with a focus on mortality data (testing particular at the beginning at the pandemic restricted to severely ill patients for diagnostic in hospitals). Sex and age distribution, at times also subgroups.	No clear goal or target formulated, instead phases are defined and an overall goal of protecting the NHS. The 3-tier, and since December 2020, the 4-tier approach clearly outlines what can be done and what is not allowed if a region or a town which goes into a certain tier. From 3 March daily press conferences aimed at explaining the government response to the COVID-19 outbreak which was interrupted on 23 June 2020 and resumed to some extent after 20 October 2020. Press conference involve the prime minister or a minister, the CMO or CSA, and most of the time an NHS representative.
Communication of uncertainty	Government included a strong narrative of uncertainty throughout the communication. The Minister of Health, Jens Spahn in a speech in the German parliament on 22 April summarised that there was a very steep joint learning curve, and that in view of all the uncertainty around the COVID-19 pandemic he foresees that we will need to apologise each other for wrong decisions.^123^	Communication of uncertainty was perceived as contra productive in view that this would lower the trust in the society.^53^ Several recommendations of the WHO and also ECDC were questioned, among them (i) contract tracing and isolating of contacts, (ii) spread of the disease by those with no symptoms (presymptomatic and asymptomatic spread), (iii) use of face-masks, (iv) border control.	Communication of uncertainty was often lacking especially in the first phase of the pandemic. Communication presented decision making as ‘following the science’, but imperfect data and uncertainty were not always communicated clearly (on personal protection equipment supply, transmission level, school transmission). Initial lack of transparency of information, including restricted SAGE meetings minutes and SAGE membership which feeds uncertainty and secrecy in decision making process.^124^

CMO, chief medical officer; CSA, Chief Scientifc Advisor; ECDC, European Centre for Disease Prevention and Control; NHS, National Health Service; RKI, Robert Koch Institute.

#### Knowledge: communication with the public

Public messaging in all three countries focused on hand hygiene and social distancing during the first wave (table 2). In view of the evolving evidence, additional messages were added in Germany such as the use of face coverings and later opening of windows in line with evolving evidence. In contrast, messages remained the same in Sweden and were limited to hygiene and social distancing. In the UK, key slogans changed multiple times in 2020 from ‘stay at home’ (March), ‘stay alert’ (May) to V-day (December). Protecting the health systems from being overwhelmed were key objectives in all three countries. Clear targets, such as an incidence rate of below 50 infections per week per 100 000 population were only set in Germany, although at a somewhat arbitrary level, but supported by modelling arguments to prevent the testing, tracing and isolating system from being overwhelmed.^51^

#### Uncertainty: dealing with uncertainty while creating trust

A particular feature of the German response was the repeated communication of uncertainty (table 2), which made it substantially easier to adapt messages over time. While the UK and Sweden claimed early on to base their approach on science and evidence, politicians in Germany repeatedly stressed they had limited information to inform their decisions: indeed, the German Health Minister Spahn expressed this in April 2020 in a speech widely reported by the media.^52^ In Sweden, the communication of uncertainty was perceived as inappropriate given that it could raise fear.^53^ The PHA repeatedly stated to base their recommendations on evidence—however, critical voices challenged this in view of the evolving evidence.^53 54^

**Figure 3:**
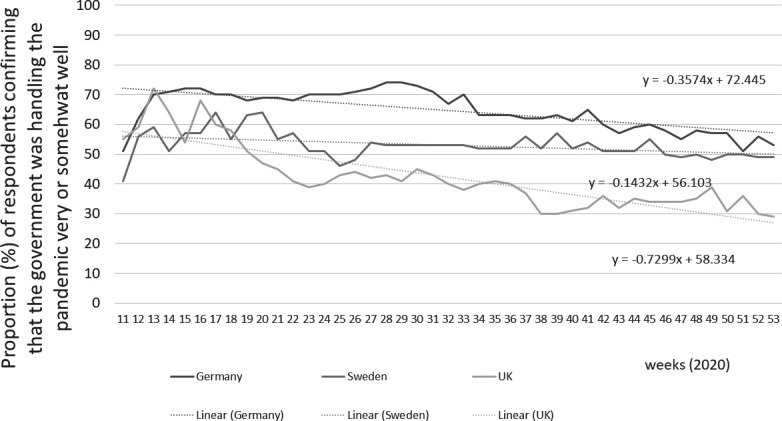
Trust in COVID-19 action by government (YouGov COVID-19 COVID tracker, missing data points imputed).

Figure 3 shows data on how populations rated their government response using the YouGov COVID-19 tracker. In all three countries, people had more confidence in their government during the first wave than the second wave. The strongest decline was in the UK (−0.73, p value for change <0.001), but trust also tailing off substantially in Germany (−0.36, p value for change <0.001). The Swedish trust figures were somewhat more stable after a very early initial decline (−0.14, p value for change 0.001). The German YouGov COVID-19 tracker trust survey broadly aligns with national polls (online supplemental file 5). Interestingly a larger share of people who were not fully happy with the government response opted for stronger restriction (consistently over 20%) than for more relaxed handling throughout 2020 (around 15%).

10.1136/bmjgh-2021-006691.supp5 Supplementary data

## Discussion

Our review of the COVID-19 responses in Germany, Sweden and the UK revealed stark differences which were linked to pre-existing governing structures, the traditional role of academia, experience of crisis management and the communication of uncertainty—all of which have an impact on how much people trusted their government. It will remain difficult to indicate clear successes and failures, however, Germany—although its prepandemic Global Health Security Index was substantially lower than that of Sweden and the UK—indicated convincing aspects of resilience: we highlight diversity and inclusiveness, the strong political and societal debate, and the largely positive involvement of the media to engage with the evolving science and the difficulties to translate science into policies. Sweden dismissed and feared plurality of voices, but trust declined less than in the other countries. The effects of the quasi-abdication of government responsibility on long-term political and social accountability will need to be seen in the future. In the UK, the strong voice of academia held governance accountable but left the public with confusing and rapidly changing public health messages.

### Governance and legitimacy of responses

Crisis management is typically characterised by a concentration of power and a shift to executive decrees.^55^ The concentration of power during the first wave was largely accepted in Germany and UK. In Sweden, similarly, polls suggest that the quasi-abdication of power to a public authority was accepted.^56^ Germany drew on its experience from several crises including the reunification in 1990 and the 2008 financial crisis.^57^ Communication and response have been described as cocreated allowing many actors to debate at national and local level.^36^ The German system seemed to profit from its devolved health systems and decision-making,^29^ although more strongly during the first wave.^29 58^ The seemingly appropriate crisis management in the first wave stands in contrast to the relatively lower score that Germany received in the 2019 Global Health Security Index^27^ compared with Sweden and the UK. This could be interpreted as ‘hard resilience is more important than planned public guidance’ as Jasanoff *et al* have suggested.^57^

In Sweden, the PHA de facto led the crisis response. Criticism of crisis management is not new in Sweden: it has followed every crisis, including the Asian Tsunami in 2004 where many Swedish people were killed.^59^ Elisabeth Åsbrink suggested in a much discussed article in March 2020 that Sweden is ‘damaged by peace’.^60^ Indeed, the Swedish exceptionalism generated great international media attention,^61^ and to date it remains unclear whether herd immunity was an intended goal of the strategy as proposed by Giesecke.^62 63^ A critical scientific review of the underlying societal and political reasons of the exceptionalism has started however^54 64^ maybe overcoming the challenge that high trust in the government hampers critical debates.^65^

In the UK, the pandemic hit a country which was preoccupied with Brexit. Crisis management was activated relatively early but largely consisted of Parliament deferring most debate and legislative power to the government with its ministries. Later in the pandemic, Parliament became more critical. For example, two House of Commons’ Committees have raised serious questions about the government’s handling of the test-and-trace policy and the lack of transparency and accountability in its use of data for decision-making.^66^ Parliamentary committees criticised the UK’s response as ‘hampered by overcentralised, poorly coordinated, and poorly communicated’ policies, the side-lining of local providers and failing to work and share data with local authorities,^66^ despite advice early on.^67^ The report also criticised austerity policies in health, with years of underfunding which left local services ill equipped to cope, as well as some technical failures.^67 68^

### Knowledge and uncertainty: academia’s role in and evidence creation

Uncertainties about the pandemic meant that policymakers needed to find ways to include scientific bodies in decision-making. Both the UK and Germany relied on existing structures. Moreover, members of Germany’s government are public health specialists and chancellor Merkel is a scientist herself. Their communications about scientific evidence acknowledged uncertainties from the beginning, and consequently the possibility that policies may need to change. In Sweden, the government relied largely on the PHA, with the state epidemiologist being dominant in media and discussion.

In the UK, uncertainty was addressed with a heavy reliance on modelling while largely dismissing its limitations.^31 69^ Diversity in shaping public opinion and influencing policies was strongly demanded by an active and vocal science community, illustrated by the creation of alternative bodies (such as Independent SAGE), and strongly worded opinion pieces and blogs and science in scientific and medical journals and the media. These highlighted the importance of academic values and transparency, although they were slow to change government approaches.^70^ However, multiple voices sometimes created confusion for the public,^71^ and government insistence that it was always right may have eroded trust in government,^72^ although this changed following the successful vaccination programme.

In Germany, TV debates are well established and were used for continuous information sharing. In Sweden, only a few TV debate sessions were facilitated. This aligns with the described hesitancy of public debate in Sweden and journalists’ relative lack of engagement into scrutiny. Several authors have highlighted the lack of media engagement might have contributed to the limited questioning of the Swedish PHA’s handling of the pandemic.^54 73–75^ This also might have contributed to the Swedish strategy considered unclear and subject to interpretation.^30 61^

### Creating trust in the population

Trust in government increased in those countries in 2020 which were able to almost eliminate COVID-19 such as Australia and New Zealand.^76^ Trust, however, is not linear, but reciprocal.^77^ Some studies indicate a correlation between trust and spread.^78^ High trust might lead to over-reliance on government, thereby decreasing personal efforts to combat the pandemic.^79^ Such over-reliance and greater trust towards the government might have led people in Sweden to take a rather relaxed approach. Mobility data suggest that there are more limited changes in Sweden compared with other European cities during the early first wave.^80^ Other studies appoint to correlations between trust and compliance.^81^ The importance of creating and continuously maintaining trust cannot be sufficiently underscored. Experience of pandemic outbreaks elsewhere shows that even in situations of poor knowledge and knowledge asymmetry, trust-building is an essential component,^82^and global efforts have sought to address this.

In all three countries, surveys suggest that the public had relatively high levels of trust in their governments at the start of the pandemic, with strong declines in Germany and the UK. The German decline was probably in response to the increasing federal misalignment referred to as a ‘patchwork quilt’ in the media. While localised actions were a clear strength in the first wave under a unified national approach,^29^ diverging targets and rules in the different states were confusing during the second wave. While a growing share of the public sought tougher measures, the government seemed to be wary of the 10%–20% of the population who were opposed—a response which probably reduced trust further.

The Swedish response has been widely described as a ‘close partnership between the government and the society based on mutual trust’.^83^ Still, trust was continuously lower than in Germany, but remained more stable. While this approach could be seen as a success,^53^ the longer-term effects of stifling an honest debate remaining unclear.

Trust in the UK declined dramatically early in the pandemic, probably associated with the strong public and academic debate, and in reaction to little transparency in government decision-making.^84^ The multiplicity of views created at times significant debate and controversy, leading to public confusion.^85^

### Relevance of the resilience framework

Our analysis underlines the relevance of our resilience framework highlighting critical government action. It highlighted the need for a balance between critical discourse, diversity, decisive action and clear communication as also recently highlighted.^86^ While the elements of the resilience framework were originally designed to explain *health systems* resilience, we had to adapt them to include state level action more strongly. We highlight processes, but we do not link the findings to any outcomes, given that the crisis is ongoing, and using measures of COVID-19 infection rates and mortality would create a short-sighted view. For instance, there is insufficient data to allow us to assess how well health systems continued to care for other diseases.^87^

The concept of resilience can be criticised. Interdependence between a crisis and long-term vulnerability is under conceptualised.^88^ The resilience framework from Blanchet *et al* explicitly aims to highlight how systems transform to create something new and better^34^ while the term resilience has been criticised by others as too static and focused on vulnerability.^89^

### Strength and limitations

We acknowledge the limitations of our review. We constructed the timeline based on published data, but subjectivity remains. We used data on cases, death and testing from trusted sources such ECDC for comparability. To allow a comparative view on citizens’ perspectives, we used an international survey.^42^ Responses aligned relatively well in Germany with national polls (online supplemental file 5). Still, simple questions such as whether the government handled the epidemic well obscure the complex reality.

To populate the tables and predefined domains, we used document review methodologies extracting data from government homepages for all three countries, complemented by information from the public health agencies of Germany and Sweden. Due to the nature of the data, it was not possible to use search engines and we had to take partly different approaches in the three countries due to the difference in the overall governance and mass media structure. Also, it was not possible to adopt common search terms as would normally be the case in identifying documents for a document analysis, partly because of the language differences between the three countries, and partly because of the language used in relation to COVID-19 evolved very rapidly through 2020.

Depth was assured by the research team living and working in the respective countries (SL in Germany, CH and JS in Sweden, SM-J, SM and NS in the UK, and were native speakers (CH and SL are German, JS Swedish, SM-J, SM and NS British). Our professional backgrounds are multidisciplinary including in health systems and policy (CH, SM-J, SM and NS) medicine (CH and SL) and governance (JS). CH and SL have a training in the control of infectious diseases. Transparency was assured by careful referencing.

## Conclusion

Our cross-country document review highlights critical aspects of governance such as establishing the role of trusted communication with the public and functioning multi-professional and independent science and advisory bodies nationally. Further, we highlight the fine balance between diversity and plurality, the power of decentralised action and the need to communicate clear and understandable goals and objectives.

Germany’s federal system and its broad societal support and academic engagement created diversity and pluralism. The localised approach might be an exemplar for a cocreated approach in a crisis—although it has hit its limits in the third wave. The UK’s engaged academic institutions informed its strategies and approaches, but the lack of transparency in government decision making undermined trust. In Sweden, high trust in the government might have hampered more critical debates.

Our hypothesis generating analysis suggests that crisis preparedness and resilience framing will need to encompass those governance structures beyond health that enable (i) strong and legitimate leadership facilitating decentralised action and (ii) trusted links to science and advisory bodies. A media structure which is prepared to communicate science and facilitate debate seams to support resilience. Cross-country learning should trump nationalism.

## Data Availability

All data relevant to the study are included in the article or uploaded as supplementary information. All information are in public spaces and referenced.
