# Supplementary material for: National health governance, science and the media: drivers of COVID-19 responses in Germany, Sweden and the UK in 2020
Source: BMJ Glob Health. 2021 Nov 17;6(12):e006691. doi: 10.1136/bmjgh-2021-006691 (PMC8764706; doi:10.1136/bmjgh-2021-006691)
Supplement: Supplementary data [file bmjgh-2021-006691supp001.pdf]

Web annex 1: List of websites searched and search term:

Germany:

Robert Koch Institute https://www.rki.de/DE/Home/homepage_node.html: Leitbild, Coronavirus, Gesundheitsmonitoring, Fallzahlen und Meldungen, Allgemeine Infektionsschutzmassnahmen, Forschung

Bundesregierung https://www.bundesregierung.de/breg-de: Coronavirus, Kabinett, Infektionsschutzgesetz

Bundesgesundheitsamt https://www.bundesgesundheitsministerium.de/: Coronavirus

ARD https://www.daserste.de/sendungen-a-z/archive-az-100.html and ZDF https://www.zdf.de/sendungen-a-z: Coronavirus

Sweden:

Public Health Agency: https://www.folkhalsomyndigheten.se/: om Folkhälsomyndikheten, uppdrag, Covid-19, Smittskydd & beredskap, statistik och analyser, Verksamheter, undersökningar och studier

Government: https://www.regeringen.se/: Coronapandemin, krisberedtskap

UK:

Government https://www.gov.uk/: Coronavirus, legislation, Coronavirus Act 2020, SAGE, test, track and trace, deaths testing for coronavirus
Parliament https://www.parliament.uk/: coronavirus, House of Commons

Independent SAGE https://www.independentsage.org/
