# Supplementary figures and images for: National health governance, science and the media: drivers of COVID-19 responses in Germany, Sweden and the UK in 2020
Source: BMJ Glob Health. 2021 Nov 17;6(12):e006691. doi: 10.1136/bmjgh-2021-006691 (PMC8764706; doi:10.1136/bmjgh-2021-006691)

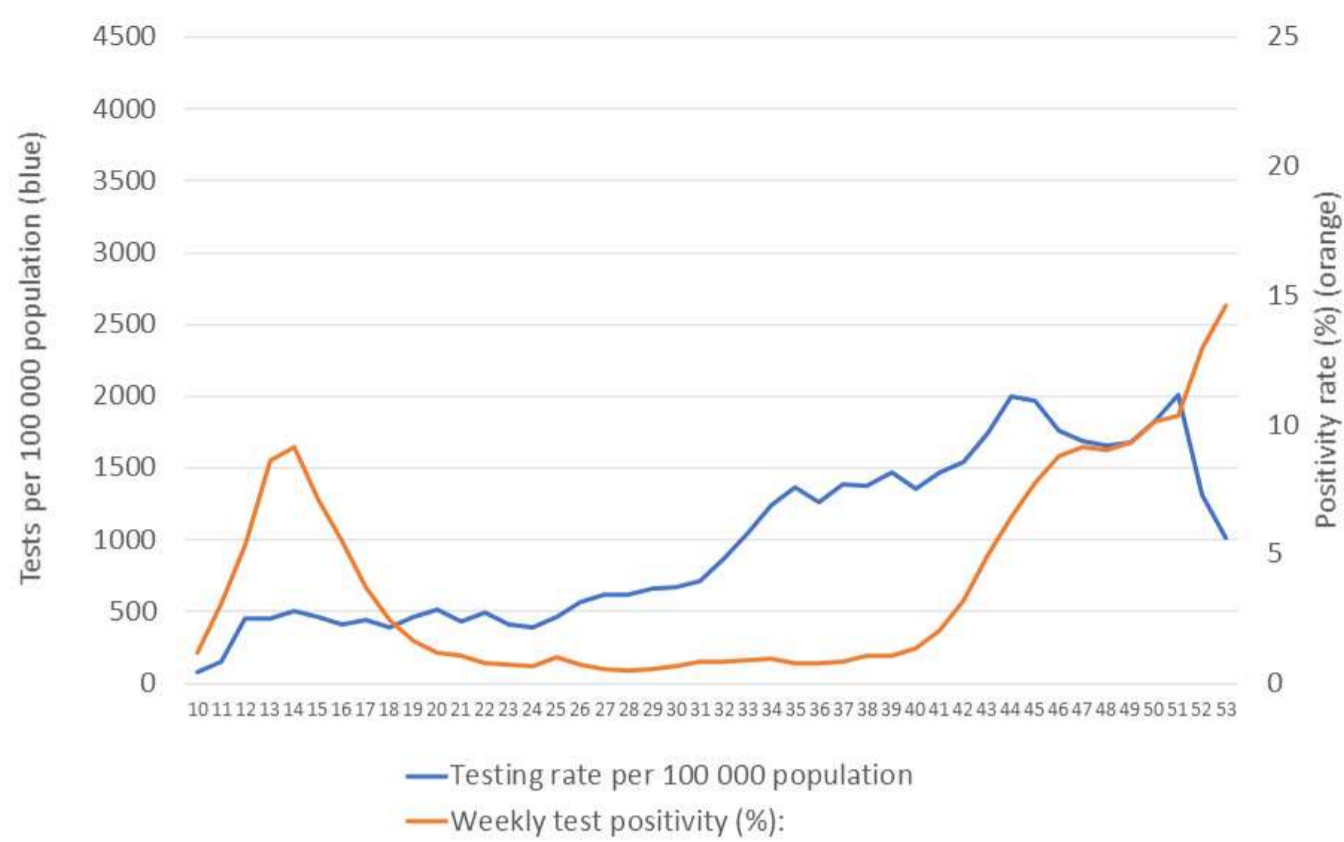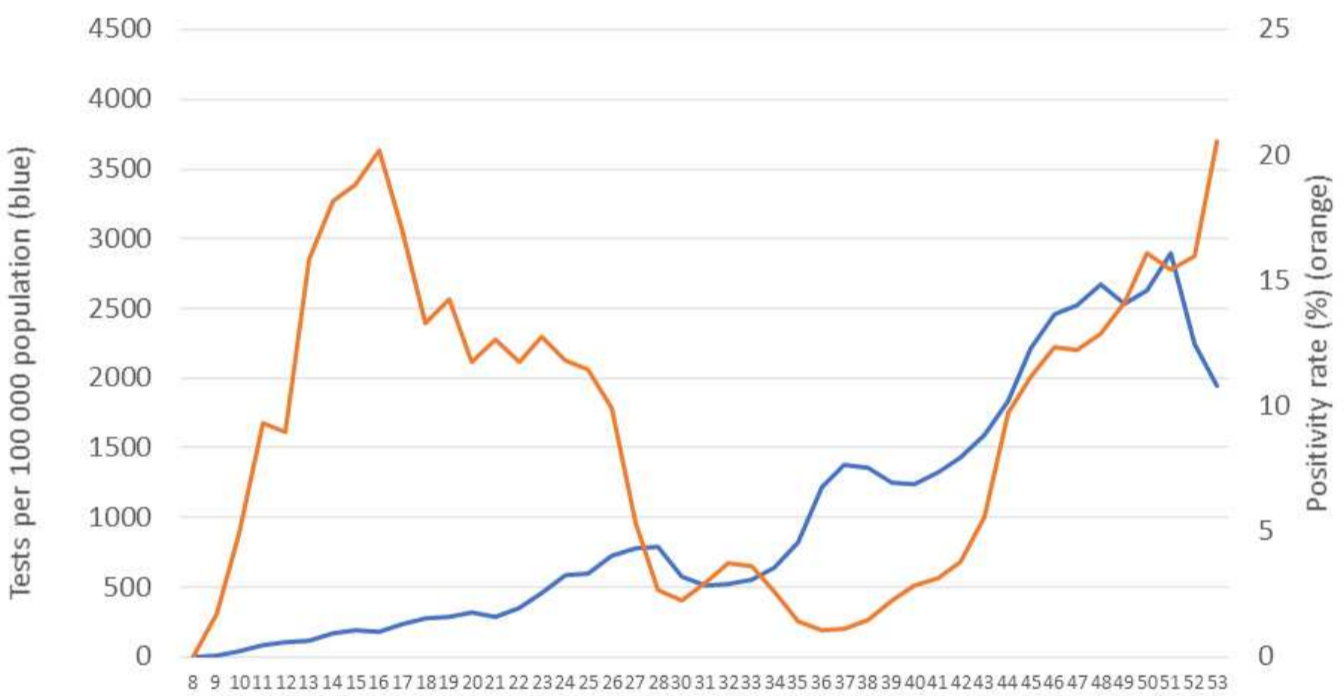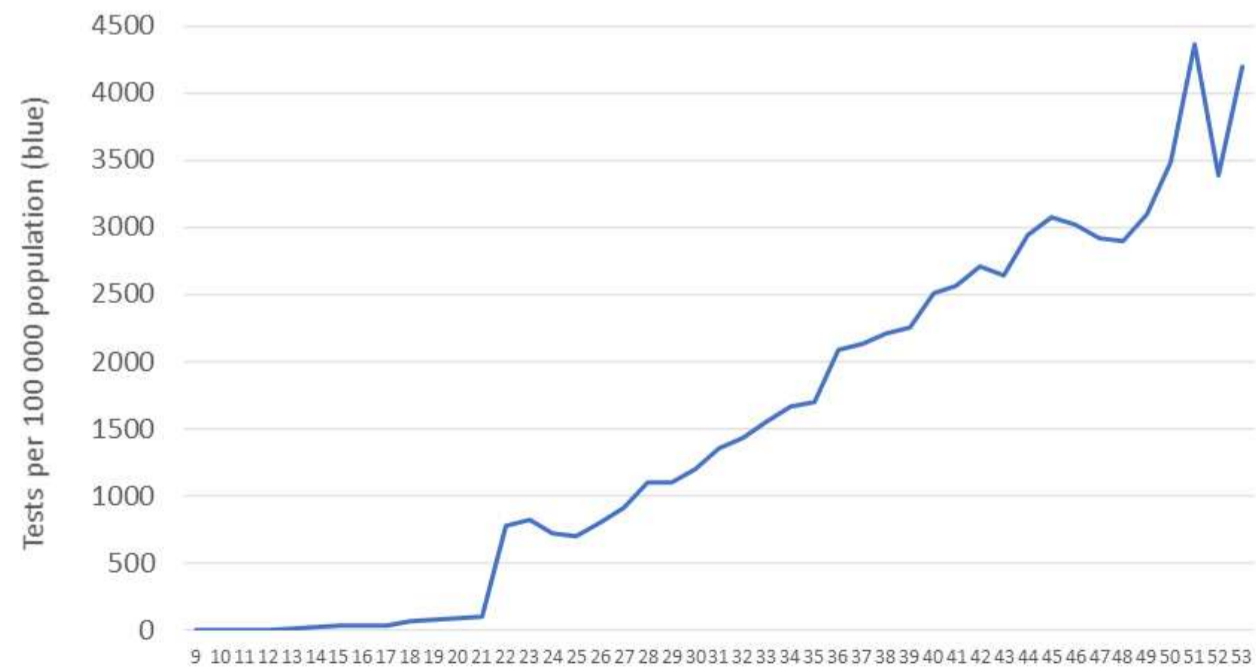

Annex Fig 1: Testing rate (blue) and positivity (yellow)

Supplement: Supplementary data [file bmjgh-2021-006691supp004.pdf]
