# Supplementary material for: National health governance, science and the media: drivers of COVID-19 responses in Germany, Sweden and the UK in 2020
Source: BMJ Glob Health. 2021 Nov 17;6(12):e006691. doi: 10.1136/bmjgh-2021-006691 (PMC8764706; doi:10.1136/bmjgh-2021-006691)
Supplement: Supplementary data [file bmjgh-2021-006691supp002.pdf]

Web Annex 2: Web annex table 1 References for timeline

Month	Germany	Sweden	UK
January / Feb	28th Jan Activation of emergency measures	31st Jan first case[1]<br>26 Feb High alert[1]	10th February they launched the Health Protection (coronavirus) Regulations.
	1st Feb Extension of the national surveillance system to include SARS-CoV-2 / COVID-19<br>28 Feb limited level of treat by RKI[2]		
March	The RKI raised the level of thread containment and follow up of cases to establish the infection threads Adapted Pandemieplan [3].<br>We stay home" became a main slogan[4]<br>9th of March: ban of larger events<br>11th March speech from Merkel[5]<br>13th March closure of schools, non-essential jobs, cultural events[2]	11 Mar gatherings of > 500 people forbidden[1]<br>16 Mar encouragement to work from home if possible[1]<br>17 Mar distance learning grade 10-12 and universities[1]<br>27 Mar gathering of > 50 people forbidden[1]<br>30 Mar visits to elderly care banned[1]	2 March SAGE proposes community transmission[6]<br>16 March Imperial college modelling study published<br>16 March advise against non-essential travelling[6]<br>18 / 20 March announcement to close schools, cafés, cultural activities etc[6]<br>23 March shutdown except essential serices and work[6]
April	Careful opening strategy [7]<br><br>22 April Schools open in some Bundesländer<br>29th April making becomes mandatory in public spaces[8]	14 April 22 scientist requests the government to take over the handling[9]<br>15 April claim of that 1/3rd of the Stockholm population to be infected by 1st May, widely picked also by international media[10,11]	6 April PM admitted to intensive care unit with COVID-19 infection[12]
May		30 May, Johan Giesecke publishes "The invisible pandemic"[13]	10 May easing of restrictions[6]
June / July	16th June Corona waning app launched[2]	Summer holidays started on the 9th of June<br>8 Juli New modelling published[14]	15 June Face masks compulsory<br>23 face masks guidance published
August	1st August Corona protests in Berlin[15]	17 Aug, schools re-open after the summer break, all classes<br>21 Aug Lövfen confirms that the strategy was right[16]	8 August further face mask restrictions come into force[12]
September	29 Sept traffic light system for regional responses agreed[17]		9th September rule of six published[12]<br>21st September pubs closed after 10 pm[12]
October	7 Oct Beherbergungsverbot [18]		12th October 3-trier system published[12]

	29 Oct partial lockdown[19]		
November		16 Nov Löfvén promises to take responsibility[20]<br>24 Nov rule of eight [21]	
December	1st Dec further restrictions to meet people[19]<br>16th December comprehensive restrictions including closing of schools and non-essential shops[22]	15 Dec Corona Commission[23]<br>Swedish King, Carl Gustaf says "I think we have failed" [24]	8th Dec Immunization campaign started[12]<br>19th Dec mutants detected[12]<br>19th 4 trier published (stay at home order)[12]

References

1. Ludvigsson JF. The first eight months of Sweden's COVID-19 strategy and the key actions and actors that were involved. *Acta paediatrica (Oslo, Norway : 1992)* 2020; **109**(12): 2459-71.
2. VDAB. Ereignis-Zeitstrahl. 2020. https://www.corona.vdab.de/zeitstrahl/?L=0 (accessed 28 March 2021).
3. Robert Koch Institute. Vorvereitungen aud Massnahmen in Deutschland, Version 1.0 (Stand 04.03.2020), Ergänzung zum Nationalen Pandemieplan – COVID-19 – neuartige Coronaviruserkrankung. 2020. https://www.rki.de/DE/Content/InfAZ/N/Neuartiges_Coronavirus/Ergaenzung_Pandemieplan_Covid.pdf?__blob=publicationFile (accessed 12 April 2020).
4. Bundesministerium fuer Gesundheit. Zusammen gegen Corona: #WirBleibenZuhause. 2020. https://twitter.com/bmg_bund/status/1240315836369440768?lang=en (accessed 8 Jan 2021).
5. Merkel: Coronavirus is Germany's greatest challenge since World War II. 2020 Deutsche Welle 2020.
6. Sanders KB. British government communication during the 2020 COVID-19 pandemic: learning from high reliability organizations. *Church, Communication and Culture* 2020; **5**(3): 356-77.
7. Safi M, Willsher K, Holmer O. Germany and Norway ease lockdonw but Spain and France hold off. *The Guardian, https://wwwtheguardiancom/world/2020/apr/19/israel-and-south-korea-to-ease-coronavirus-lockdowns* 2020.
8. Deutschland.de. Coronavirus in Germany 2020. https://www.deutschland.de/de/die-timeline-coronavirus-germany-deutschland (accessed 28 March 2021).
9. 22 Forskare. Folkhälsomyndigheten har misslyckats - nu måste politikerna gripa in. Dagens Nyheter. 14 April 2020.
10. Public Health Agency. Skattning av peakdag och antal infekterade i covid-19-utbrottet i Stockholms län februari-april 2020 2020. https://www.folkhalsomyndigheten.se/publicerat-material/publikationsarkiv/s/skattning-av-peakdag-och-antal-infekterade-i-covid-19-utbrottet-i-stockholms-lan-februari-april-2020/ (accessed 15 Feb 2021).
11. Nikel D. Sweden: 600,000 Coronavirus Infections In Stockholm By May 1, Model Estimates 2020, Forbes Apr 21,. https://www.forbes.com/sites/davidnikel/2020/04/21/sweden-600000-coronavirus-infections-in-stockholm-by-may-1-model-estimates/ (accessed 2 Jan 2021).
12. British Foreign Policy Group. COVID-19 timeline. 2020. https://bfpg.co.uk/2020/04/covid-19-timeline/ (accessed 28 March 2021).
13. Giesecke J. The invisible pandemic. *Lancet (London, England)* 2020; **395**(10238): e98.
14. Public Health Agency. Scenarier – Tre smittspridningsscenarier inom regeringsuppdraget ”Plan inför eventuella nya utbrott av covid-19” 2020. https://www.folkhalsomyndigheten.se/publicerat-material/publikationsarkiv/s/scenarier--tre-smittspridningsscenarier-inom-regeringsuppdraget-plan-infor-eventuella-nya-utbrott-av-covid-19/ (accessed 15 Feb 2021).

15. Coronavirus: Thousands protest in Germany against restrictions. https://www.bbc.com/news/world-europe-53622797 (accessed 28 March 2021).
16. Löfven S. Löfven om Sveriges strategi: Vi valde rätt. Expressen, . 2020.
17. Bundesgesundheitsministerium. Chronik-Coronavirus. 2020. https://www.bundesgesundheitsministerium.de/coronavirus/chronik-coronavirus.html (accessed 28 March 2021).
18. zdfheute. Beherbergungsverbot: Fast alle Länder einig 7 Oct 2020. https://www.zdf.de/nachrichten/politik/coronavirus-beherbergungsverbot-reisende-risikogebiete-100.html (accessed 28 March 2021).
19. Zdfheute. Lockdown. Die neuen Corona-Regels von Bund und Ländern 29 October 2020. https://www.zdf.de/nachrichten/politik/coronavirus-lockdown-massnahmen-november-100.html (accessed 28 March 2021).
20. Löfven S. Presskonferens 16 Nov 2020. 2020. https://www.svt.se/nyheter/inrikes/stefan-lofven-kallar-till-presskonferens (accessed 15 Feb 2021).
21. Regeringen. Förbud mot att hålla allmänna sammankomster och offentliga tillställningar med fler än åtta deltagare. 16 Nov 2020. https://www.regeringen.se/rattsliga-dokument/departementsserien-och-promemorior/2020/11/forbud-mot-att-halla-allmanna-sammankomster-och-offentliga-tillstallningar-med-fler-an-atta-deltagare/ (accessed 15 Feb 2021).
22. Zdfheute. Die Dezember-Beschluesse im Ueberblick 13 December 2020. https://www.zdf.de/nachrichten/politik/corona-shutdown-einzelhandel-schulen-kitas-100.html (accessed 28 March 2021).
23. Melin M, Ahlbäck Öberg S, A E, et al. The Corona Commission. Elderly care during the pandemic. Ministry of Health and Social Affairs, summary of SOU 2020:80. 2020. https://www.government.se/4af26a/contentassets/2b394e1186714875bf29991b4552b374/summary-of-sou-2020_80-elderly-care-during-the-pandemic.pdf (accessed 2 Jan 2021).
24. Lundahl P. King speaks out on pandemic: "We have failed". 17 Dec 2020. https://sverigesradio.se/artikel/7626948 (accessed 15 Feb 2021).
