# Supplementary material for: National health governance, science and the media: drivers of COVID-19 responses in Germany, Sweden and the UK in 2020
Source: BMJ Glob Health. 2021 Nov 17;6(12):e006691. doi: 10.1136/bmjgh-2021-006691 (PMC8764706; doi:10.1136/bmjgh-2021-006691)
Supplement: Supplementary data [file bmjgh-2021-006691supp003.pdf]

Web annex 4 Web annex table 2: Media debates with Covid-19 as the main topic

Weeks analysed	**Germany** main public channels: ARD and ZDF, selected search for the second week per month	**Sweden**<br>Two public channels SVT 1 & 2
9-15 March 2020	A. Wills (ARD)<br>M. Illner (ZDF)<br>M. Lanz, 2 sessions (ZDF)	SVT has the debate format Mötet where in 2020 COVID-19 was 7x featured:<br><br>Framtiden, Forskana, Kulturen, Kostnaden, Karantän, Välfärden, Debatten
6-12 April 2020	M. Illner (ZDF)<br>M. Lanz, 2 sessions (ZDF)	
11-16 May 2020	A Wills (ARD)<br>M. Illner (ZDF)<br>M Lanz 2 sessions(ZDF)	
8-14 June 2020	M. Illner (ZDF)<br>M Lanz (ZDF)	
6-12 July 2020	Sommerbreak	
10-16 August 2020	Sommerbreak	
7-13 Sept 2020	S. Maischberger (ARD)<br>M. Illner (ZDF)<br>M Lanz, 3 sessions (ZDF)	
12-18 Oct	S. Maischberger (ARD)<br>M. Illner (ZDF)<br>M. Lanz, 3 sessions (ZDF)	
9-15 Nov	A Wills (ARD)<br>S. Maischberger (ARD)<br>M. Illner (ZDF)<br>M Lanz, 3 sessions (ZDF)	
7-13 Dec 2020	S. Maischberger (ARD)<br>M. Illner (ZDF)<br>M Lanz, 3 sessions (ZDF)	

https://www.zdf.de/suche?q=illner&synth=true&sender=Gesamtes+Angebot&from=2020-06-08T00%3A00%3A00.000Z&to=2020-06-14T00%3A00%3A00.000Z&attrs=&abName=ab-2021-08-16&abGroup=gruppe-b

https://daserste.ndr.de/annewill/archiv/erste318_glossaryPage-7.html

https://www.svtplay.se/sok?q=CORONA
