# Supplementary material for: National health governance, science and the media: drivers of COVID-19 responses in Germany, Sweden and the UK in 2020
Source: BMJ Glob Health. 2021 Nov 17;6(12):e006691. doi: 10.1136/bmjgh-2021-006691 (PMC8764706; doi:10.1136/bmjgh-2021-006691)
Supplement: Supplementary data [file bmjgh-2021-006691supp005.pdf]

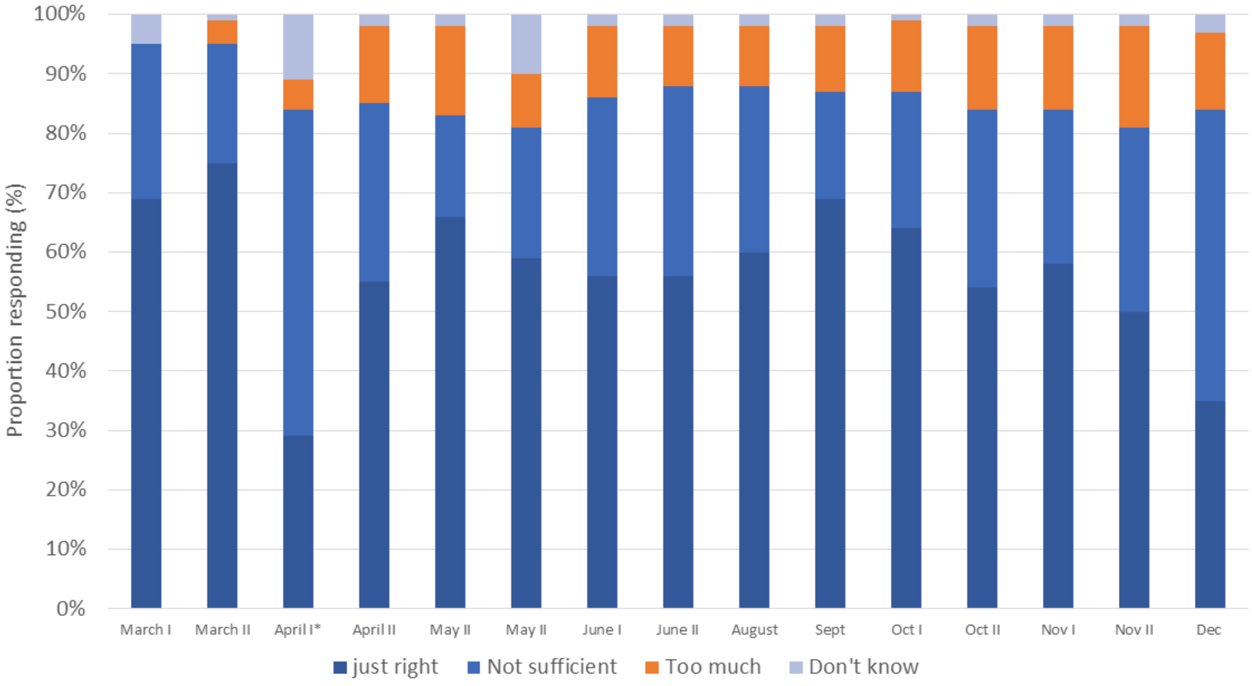

Web annex Figure 2: German polls of how the population views the Corona measures
https://www.forschungsgruppe.de/Umfragen/Politbarometer/Archiv/Politbarometer_2020/
